# Supplementary material for: Supramolecular Hybrids from Cyanometallate Complexes and Diblock Copolypeptide Amphiphiles in Water
Source: Molecules. 2022 May 19;27(10):3262. doi: 10.3390/molecules27103262 (PMC9143414; doi:10.3390/molecules27103262)
Supplement: Supplementary file 1 [file molecules-27-03262-s001.zip › molecules-1711170-supplementary.pdf]

molecules

MDPI

Supplementary Materials

Supramolecular Hybrids from Cyanometallate Complexes and Diblock Copolypeptide Amphiphiles in Water

Takayuki Tanaka[1], Keita Kuroiwa[1*]

[1] Department of Nanoscience, Faculty of Engineering, Sojo University, 4-22-1 Ikeda, Nishi-ku, Kumamoto 860-0082, Japan;

* Author to whom correspondence should be addressed; E-Mail: keitak@nano.sojo-u.ac.jp; Tel./Fax: +81-96-326-3891.

1. ^{1}H NMR spectra of copolypeptide amphiphiles

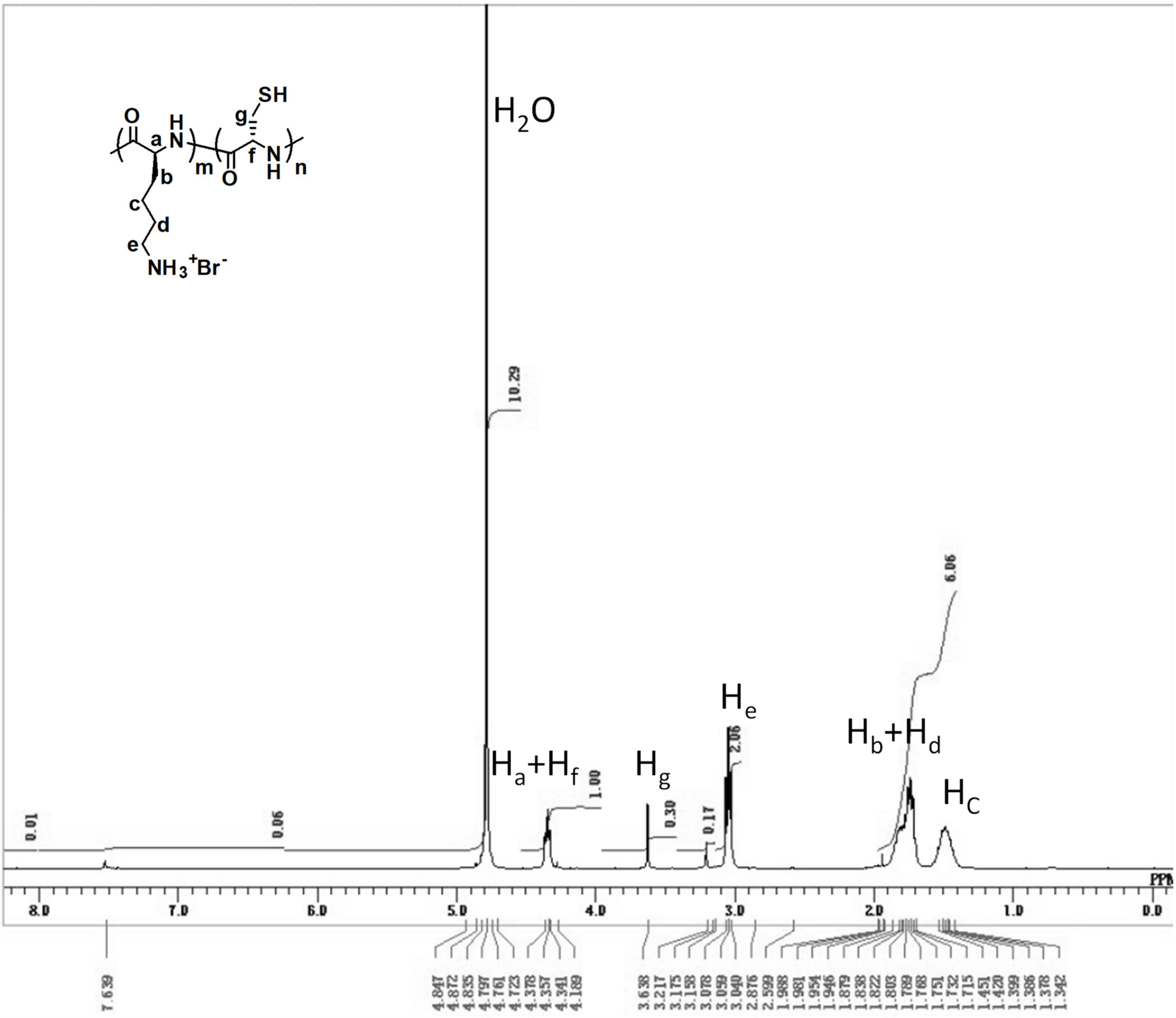

Figure S1. ^{1}H NMR spectrum of Lys$_{390}$-*b*-Cys$_4$ (**1**) (400 MHz, D_2O, r.t.).

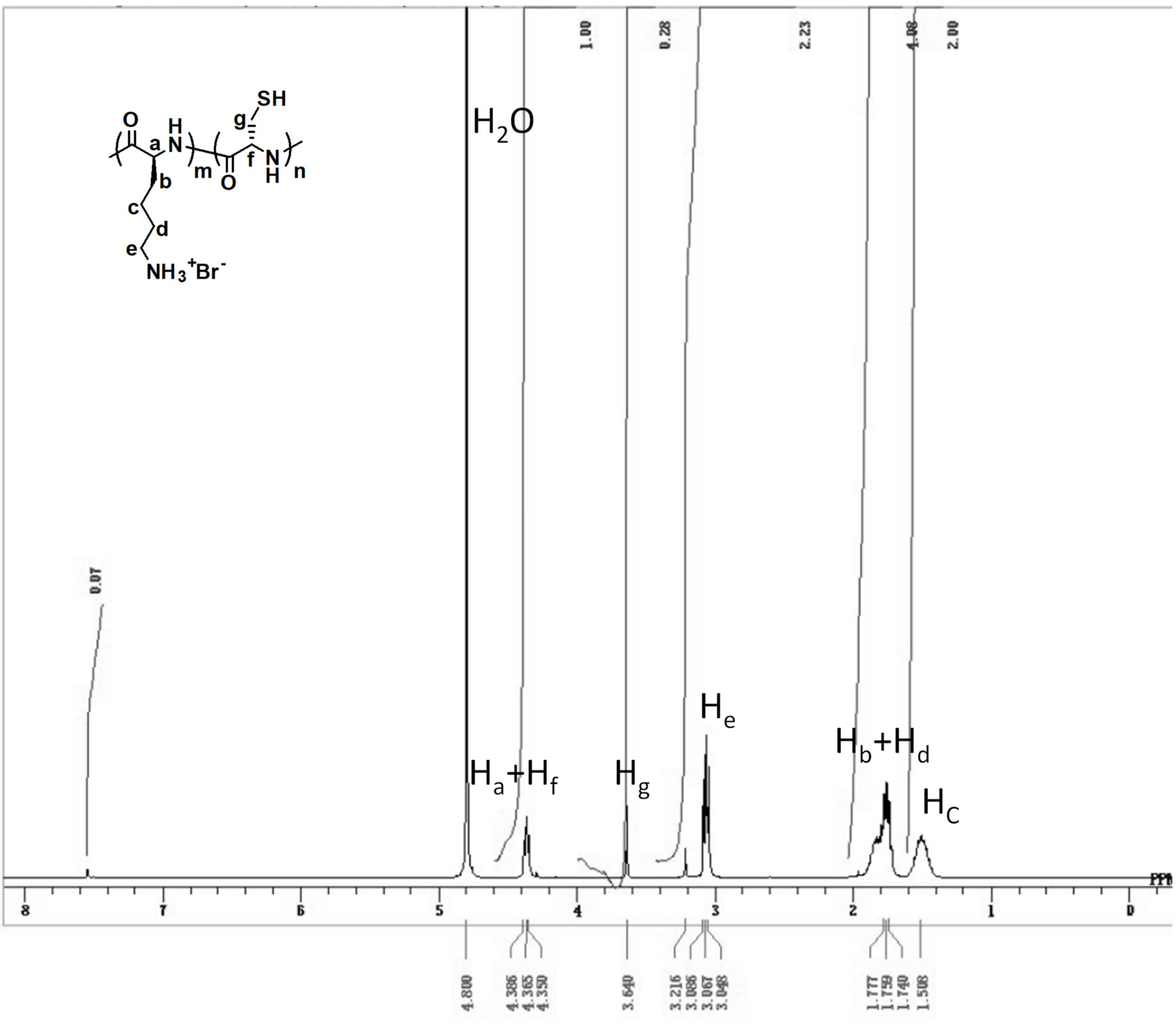

Figure S2. ^{1}H NMR spectrum of Lys_{218}-*b*-Cys_4 (**2**) (400 MHz, D_2O, r.t.).

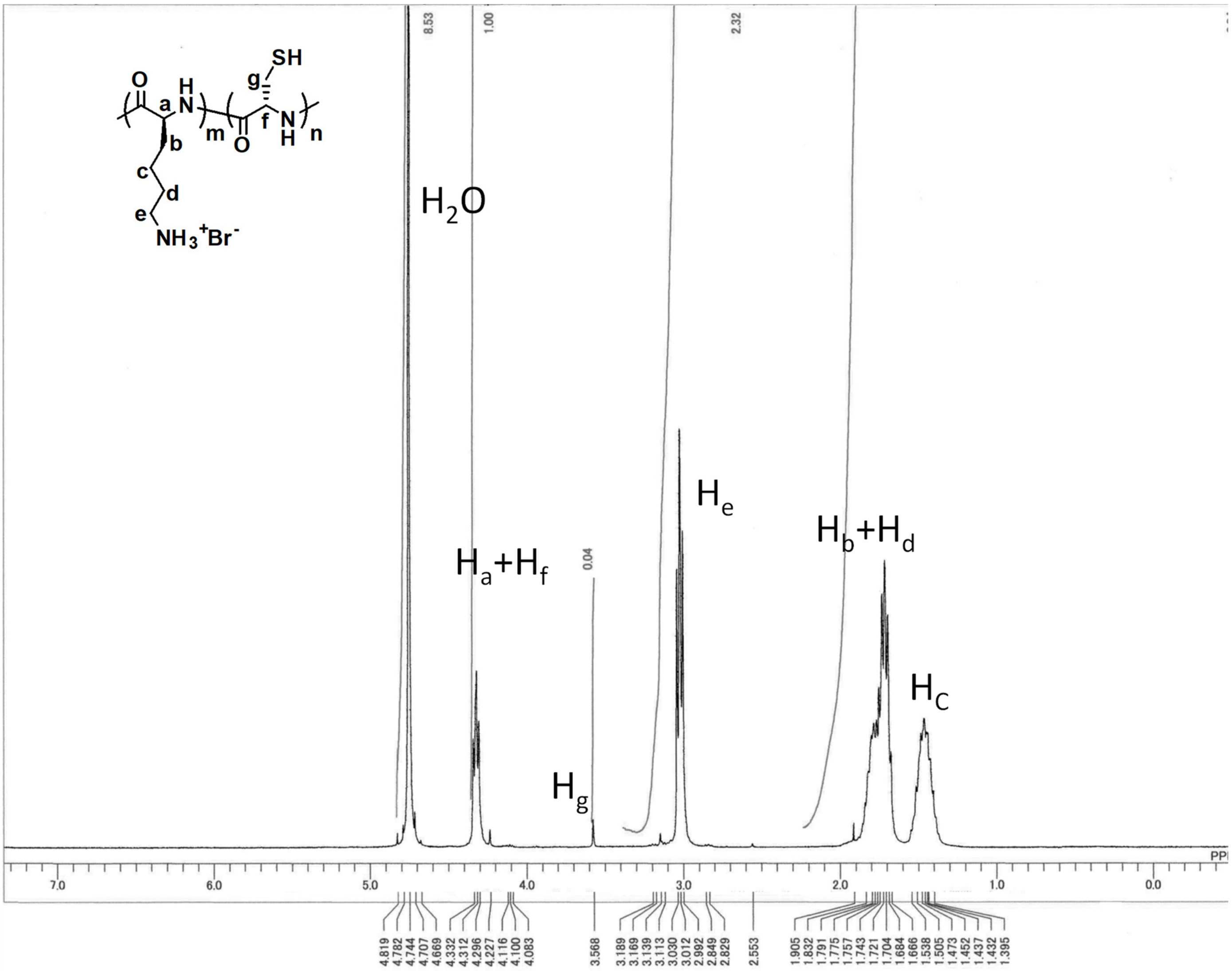

Figure S3. ^{1}H NMR spectrum of Lys$_{206}$-*b*-Cys$_4$ (**3**) (400 MHz, D_2O, r.t.).

2. IR spectra of copolypeptide amphiphiles

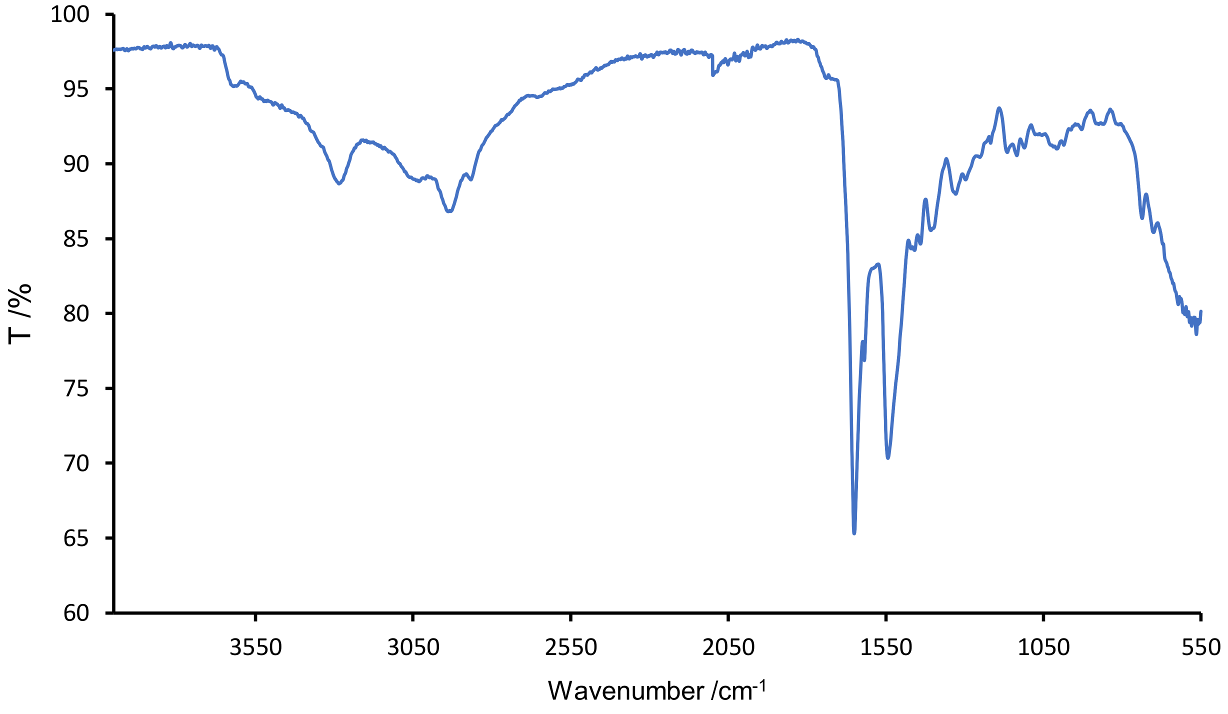

Figure S4. IR spectrum of Lys$_{390}$-*b*-Cys$_4$ (**1**) (ATR).

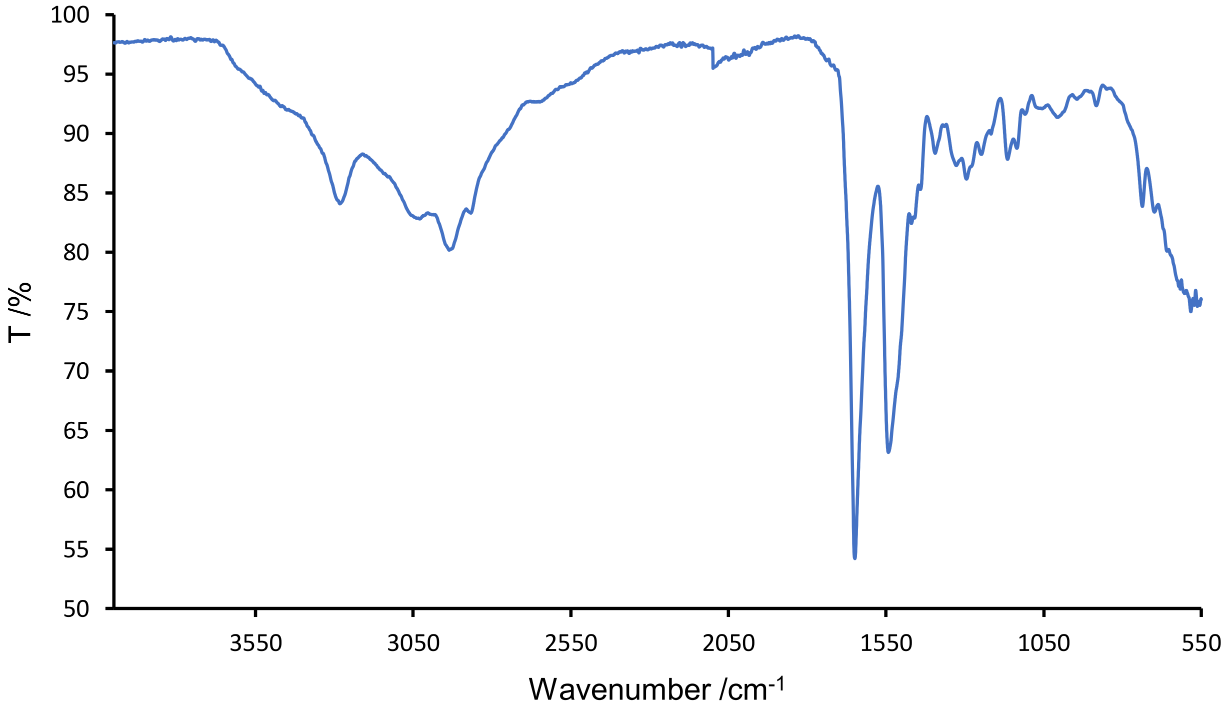

Figure S5. IR spectrum of Lys$_{218}$-*b*-Cys$_4$ (**2**) (ATR).

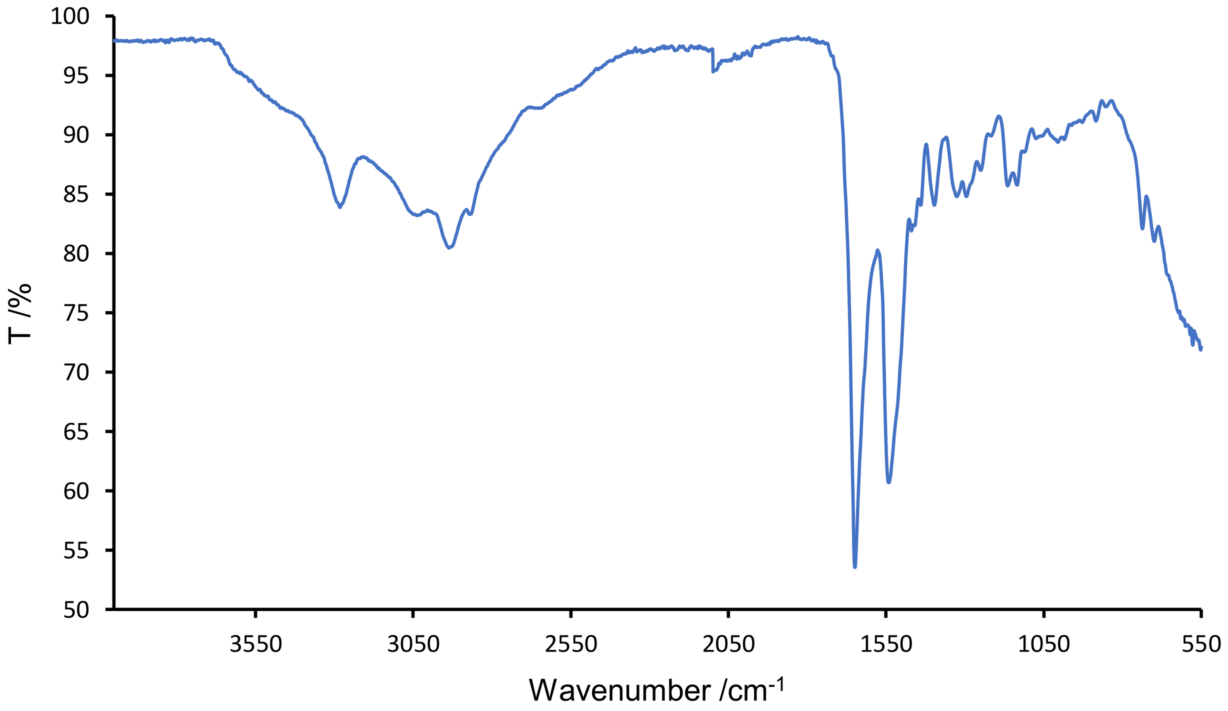

Figure S6. IR spectrum of Lys$_{206}$-*b*-Cys$_4$ (**3**) (ATR).
